# Integration of Artificial Intelligence Decision Aids to Reduce Workload and Enhance Efficiency in Thyroid Nodule Management

**DOI:** 10.1001/jamanetworkopen.2023.13674

**Published:** 2023-05-16

**Authors:** Wen-Juan Tong, Shao-Hong Wu, Mei-Qing Cheng, Hui Huang, Jin-Yu Liang, Chao-Qun Li, Huan-Ling Guo, Dan-Ni He, Yi-Hao Liu, Han Xiao, Hang-Tong Hu, Si-Min Ruan, Ming-De Li, Ming-De Lu, Wei Wang

**Affiliations:** 1 Department of Medical Ultrasonics, Institute of Diagnostic and Interventional Ultrasound, Ultrasomics Artificial Intelligence X-Lab, The First Affiliated Hospital, Sun Yat-sen University, Guangzhou, China; 2 Department of Medical Ultrasonics, The Seventh Affiliated Hospital, Sun Yat-sen University, Shenzhen, China; 3 Clinical Trials Unit, The First Affiliated Hospital, Sun Yat-sen University, Guangzhou, China; 4 Department of Hepatobiliary Surgery, The First Affiliated Hospital, Sun Yat-sen University, Guangzhou, China

## Abstract

**Importance:**

To optimize the integration of artificial intelligence (AI) decision aids and reduce workload in thyroid nodule management, it is critical to incorporate personalized AI into the decision-making processes of radiologists with varying levels of expertise.

**Objective:**

To develop an optimized integration of AI decision aids for reducing radiologists’ workload while maintaining diagnostic performance compared with traditional AI-assisted strategy.

**Design, Setting, and Participants:**

In this diagnostic study, a retrospective set of 1754 ultrasonographic images of 1048 patients with 1754 thyroid nodules from July 1, 2018, to July 31, 2019, was used to build an optimized strategy based on how 16 junior and senior radiologists incorporated AI-assisted diagnosis results with different image features. In the prospective set of this diagnostic study, 300 ultrasonographic images of 268 patients with 300 thyroid nodules from May 1 to December 31, 2021, were used to compare the optimized strategy with the traditional all-AI strategy in terms of diagnostic performance and workload reduction. Data analyses were completed in September 2022.

**Main Outcomes and Measures:**

The retrospective set of images was used to develop an optimized integration of AI decision aids for junior and senior radiologists based on the selection of AI-assisted significant or nonsignificant features. In the prospective set of images, the diagnostic performance, time-based cost, and assisted diagnosis were compared between the optimized strategy and the traditional all-AI strategy.

**Results:**

The retrospective set included 1754 ultrasonographic images from 1048 patients (mean [SD] age, 42.1 [13.2] years; 749 women [71.5%]) with 1754 thyroid nodules (mean [SD] size, 16.4 [10.6] mm); 748 nodules (42.6%) were benign, and 1006 (57.4%) were malignant. The prospective set included 300 ultrasonographic images from 268 patients (mean [SD] age, 41.7 [14.1] years; 194 women [72.4%]) with 300 thyroid nodules (mean [SD] size, 17.2 [6.8] mm); 125 nodules (41.7%) were benign, and 175 (58.3%) were malignant. For junior radiologists, the ultrasonographic features that were not improved by AI assistance included cystic or almost completely cystic nodules, anechoic nodules, spongiform nodules, and nodules smaller than 5 mm, whereas for senior radiologists the features that were not improved by AI assistance were cystic or almost completely cystic nodules, anechoic nodules, spongiform nodules, very hypoechoic nodules, nodules taller than wide, lobulated or irregular nodules, and extrathyroidal extension. Compared with the traditional all-AI strategy, the optimized strategy was associated with increased mean task completion times for junior radiologists (reader 11, from 15.2 seconds [95% CI, 13.2-17.2 seconds] to 19.4 seconds [95% CI, 15.6-23.3 seconds]; reader 12, from 12.7 seconds [95% CI, 11.4-13.9 seconds] to 15.6 seconds [95% CI, 13.6-17.7 seconds]), but shorter times for senior radiologists (reader 14, from 19.4 seconds [95% CI, 18.1-20.7 seconds] to 16.8 seconds [95% CI, 15.3-18.3 seconds]; reader 16, from 12.5 seconds [95% CI, 12.1-12.9 seconds] to 10.0 seconds [95% CI, 9.5-10.5 seconds]). There was no significant difference in sensitivity (range, 91%-100%) or specificity (range, 94%-98%) between the 2 strategies for readers 11 to 16.

**Conclusions and Relevance:**

This diagnostic study suggests that an optimized AI strategy in thyroid nodule management may reduce diagnostic time-based costs without sacrificing diagnostic accuracy for senior radiologists, while the traditional all-AI strategy may still be more beneficial for junior radiologists.

## Key Points

**Question:**

How can the integration of artificial intelligence (AI) decision aids be optimized to enhance efficiency in thyroid nodule management?

**Findings:**

In this diagnostic study involving 16 radiologists and 2054 ultrasonographic images, junior radiologists were instructed to use the traditional all-AI strategy, while senior radiologists were advised to apply the optimized integration of AI. The optimized approach was associated with reduced diagnostic time and maintained high diagnostic performance.

**Meaning:**

This study suggests that radiologists with different expertise levels can fully integrate AI-generated suggestions based on the optimized integration of AI decision aids, which provides the theoretical basis for human-machine integration for thyroid nodule management.

## Teaser

This diagnostic study describes the development of an optimized integration of artificial intelligence decision aids for reducing radiologists’ workload while maintaining diagnostic performance compared with traditional artificial intelligence–assisted strategy.

## Introduction

Thyroid cancer is responsible for 586 000 cases of malignant neoplasm worldwide, ranks ninth for incidence, and is the most rapidly increasing malignant neoplasm in all populations.^1^ Ultrasonography serves as the only effective imaging modality in thyroid screening settings.^2^ As the volume of examinations increases and the time for interpretation becomes longer, radiologists are under increasing pressure to deliver effective and timely diagnoses.

Currently, deep learning–based artificial intelligence (AI) using digital imaging data has shown value in thyroid nodule management by improving ultrasonographic diagnostic performance. Newly published studies demonstrated that the diagnostic performance of AI was comparable to that of radiologists, ranging from 73% to 94% for sensitivity and from 81% to 91% for specificity.^3,4,5,6^ Furthermore, the diagnostic performance of radiologists can be significantly improved with the assistance of AI, with the area under the receiver operating characteristic curve increasing from 0.837 to 0.875.^4^ The increase in popularity of AI in the management of thyroid nodules has elicited interest in clinical decision aids and improving workflow efficiency.

Two issues remain unaddressed in the loop of human-machine interaction for thyroid nodule management. First, the adoption of an AI system by radiologists and its potential clinical benefits depend largely on the level of trust radiologists have in the system’s recommendations.^7^ However, despite the potential benefits, the interaction between AI-based clinical decision-support systems and their users, such as radiologists, is poorly understood.^8^ Responses can vary depending on the task at hand or the person’s expertise at the task.^8,9^ It is important to study how radiologists with different levels of expertise will perceive and integrate AI-generated advice before such systems are deployed. Second, a major drawback of reported AI decision aids is the necessity for the radiologist to evaluate all thyroid images. The traditional AI-assisted diagnosis in double reading mode has been reported to fail to reduce radiologists’ workload or working time, and, in some cases, it has been shown to increase the time required for deliberation as well as the false-positive or false-negative rate.^10^ In contrast, some studies have explored AI-powered triaging approaches for breast cancer screening, in which examinations with a high probability of having cancer-free results are triaged and only the remaining examinations are referred to radiologists, which has the potential to alleviate their workload.^11,12^ However, to our knowledge, there is limited research on how to reduce the workload and enhance the efficiency of thyroid nodule screening.

In this study, we first retrospectively investigated the imaging features associated with the effectiveness of AI assistance to build an optimized integration of AI decision aids for 10 radiologists with different expertise levels. Then, we tested the optimized strategy in a prospective cohort of 6 radiologists to explore how to reduce screening workload while maintaining diagnostic performance.

## Methods

### Study Design and Overview

This diagnostic study was approved by the research ethics committee of the First Affiliated Hospital of Sun Yat-sen University, with a waiver of informed consent as the data were deidentified to protect the privacy of the participants. The study followed the Standards for Reporting of Diagnostic Accuracy (STARD) reporting guideline.

We first used our AI model to compare AI-assisted diagnoses by radiologists of different image features through a retrospective set study, and we identified significant and nonsignificant AI-assisted features to develop an optimized strategy. Next, we compared the differences in diagnostic performance and time-based cost between the traditional AI strategy and the optimized AI strategy through prospective set testing.

### Data Collection

For the retrospective set, 1754 ultrasonographic images of 1048 patients were retrospectively collected from the First Affiliated Hospital of Sun Yat-sen University and the Third Affiliated Hospital of Sun Yat-sen University, Guangzhou, China, between July 1, 2018, and July 31, 2019. For the prospective set, 300 ultrasonographic images of 268 patients were prospectively and consecutively collected from the First Affiliated Hospital of Sun Yat-sen University, Guangzhou, China, between May 1 and December 31, 2021. Patients aged 18 years or older with thyroid nodules detected by ultrasonography who obtained a definitive benign or malignant pathologic result (surgical specimen or fine-needle aspiration [Bethesda category II or VI]) were eligible for inclusion. Patients with low-quality ultrasonographic images (eg, with severe artifacts or a low-resolution image) were excluded.

We used various models of ultrasonographic equipment produced by 10 different manufacturers (Philips, Toshiba, Siemens, Vinno, Hitachi, Aloka, GE Healthcare, Supersonic, Mindray, and Esaote) to generate the ultrasonographic images. All thyroid ultrasonographic images extracted from the thyroid imaging database were converted into a JPEG format. All images were resized to 224 × 224 pixels.

### Building an Optimized Strategy in a Retrospective Set

For AI assistance to be effective, it must be able to make clinical decisions in indecisive situations, and radiologists must have confidence that the AI will outperform them. However, relying on AI without considering its limitations can bring its trustworthiness into doubt. To mitigate this trust issue, a human-machine integration approach is necessary. In this study, we aimed to formulate an optimized strategy that considered the role of AI assistance in junior and senior radiologists’ workload with respect to various image features, to establish positive progression in the loop of human-machine interaction. We investigated the image features, including American College of Radiology (ACR) Thyroid Imaging Reporting and Data System (TI-RADS) features,^13^ size of nodules, and parenchymal backgrounds. By comparing the diagnostic sensitivity or specificity of images with various features without or with AI assistance, we conducted statistical analysis to inform radiologists with different levels of expertise. Significant improvement in sensitivity or specificity indicated that the enhancement in AI-assisted performance was associated with these features (termed *significant feature*), and AI assistance was encouraged in such scenarios. Conversely, when there was no significant improvement, it implied that AI assistance might not be helpful in these features (termed *nonsignificant feature*). Therefore, an independent diagnosis by radiologists was recommended as long as these features were present.

In a retrospective set, a total of 1754 ultrasonographic images were characterized by 10 radiologists blinded to the pathologic result, including 5 junior radiologists (readers 1-5) and 5 senior radiologists (readers 6-10), who participated only in the retrospective set (the basic information and the definitions of junior and senior radiologists are detailed in eTable 1 and the eMethods in Supplement 1). In this set, a 3-step test was performed during the same period for each image: (1) each radiologist evaluated the image features, including ACR TI-RADS signs and thyroid parenchymal background^13^; (2) radiologists made an independent diagnosis, without the help of AI, and categorized the nodule as benign or malignant; and (3) radiologists made a secondary evaluation, based on AI recommendations, and assessed the nodule as benign or malignant. The deep learning AI model that we used was established in a previous study,^4^ and in-depth information about the deep learning AI model and its training and validationare presented in the eMethods in Supplement 1.

Thyroid parenchymal background included the homogeneous and heterogeneous parenchymal background. The nodule size had been recorded already. To assess all the features of each image, including ACR TI-RADS features and parenchymal backgrounds, all images were initially reviewed by all 10 radiologists. The final feature was assessed based on the highest proportion of agreement among the 10 radiologists. In case of a tie, the senior radiologists made the final decision. We compared the performance of an independent diagnosis and the performance of a traditional all-AI strategy diagnosis among junior and senior radiologists for a cluster of ultrasonographic images, and we identified the significant and nonsignificant features of the images associated with radiologists’ decision-making.

According to this analysis, the optimized strategy was established to guide radiologists to selectively apply AI suggestions. When significant features appeared within the images, AI assistance was encouraged; as long as nonsignificant features were observed, the radiologists’ independent diagnoses were recommended.

### Testing the Optimized Strategy in a Prospective Set

**Figure 1.**
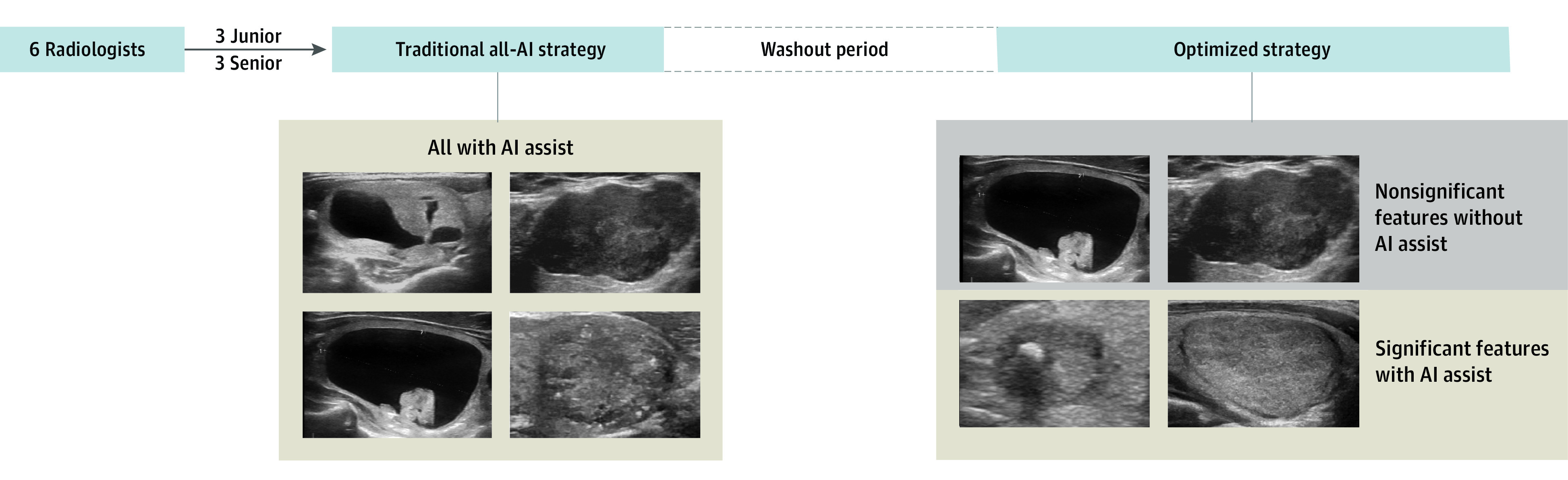
Prospective Study Profile The radiologists performed a traditional all–artificial intelligence (AI) strategy test followed by an optimized strategy test with a 4-week washout period. In the traditional all-AI strategy, radiologists were recommended to diagnose all images with AI assistance. When testing the optimized strategy, all radiologists used AI-assisted diagnosis for images with corresponding significant features; AI-assisted diagnosis was not used for images with any nonsignificant features.

Based on the optimized strategy, we used a prospective cohort to explore how to reduce screening workloads while maintaining diagnostic performance. An additional 300 prospectively collected images were tested by 6 other radiologists, including 3 junior radiologists (readers 11-13) and 3 senior radiologists (readers 14-16) (eTable 1 in Supplement 1), who participated only in the prospective set. This test was conducted during 2 periods. In the first period, radiologists assessed each nodule of 300 images based on AI recommendations and made a diagnosis of malignant or benign after evaluation of features, which was the traditional all-AI strategy diagnosis. After a 4-week washout period, the image sequence was randomly shuffled, and the radiologists made a diagnosis for each nodule according to the optimized strategy after evaluating the image features. In the optimized strategy, an AI-assisted diagnosis was provided only for images with corresponding significant features. For all images with any nonsignificant features, an AI-assisted diagnosis was not provided to senior and junior radiologists (Figure 1).

Before the start of each period, each radiologist was required to keep the strategy’s mechanism in mind and then read 20 ultrasonographic images (not belonging to the retrospective or prospective set) to become familiar with the AI and recording system. Each radiologist was eventually assigned to read all 300 ultrasonographic images. The process of image interpretation was the same as that for the retrospective set. The time-based cost was recorded from the evaluation of the features to the diagnosis of benign or malignant nodule for each image. The diagnostic performance and time-based cost were compared between the traditional all-AI strategy and the optimized strategy for junior and senior radiologists.

### Statistical Analysis

All statistical analyses were performed using SPSS, version 23.0 (IBM Corp) and MedCalc, version 15.0 (MedCalc Software). We used the McNemar test to compare the diagnostic performance (sensitivity and specificity with 95% CIs) between the optimized strategy (ie, independent diagnosis without AI) and the traditional all-AI strategy. Paired-sample *t* tests were used to compare the time-based costs at the point of difference between the traditional all-AI strategy and the optimized strategy. The diagnostic time-based cost was expressed as mean values and 95% CIs. All *P* values were from 2-sided tests and results were deemed statistically significant at *P* < .05.

## Results

### Patient Characteristics and Image Features

In the retrospective set, we analyzed 1754 images from 1048 patients (mean [SD] age, 42.1 [13.2] years; 299 men and 749 women) with 1754 thyroid nodules (mean [SD] size, 16.4 [10.6] mm) enrolled from 2 clinical sites. Of these nodules, 748 (42.6%) were benign, and 1006 (57.4%) were malignant. In the prospective set, we used 300 images from 268 patients (mean [SD] age, 41.7 [14.1] years; 74 men and 194 women) with 300 thyroid nodules (mean [SD] size, 17.2 [6.8] mm) enrolled from 1 clinical site to test the feasibility of the optimized strategy; 125 nodules (41.7%) were benign, and 175 (58.3%) were malignant. All nodules were confirmed based on pathologic findings after fine-needle aspiration or surgical resection. The demographic characteristics of the patients, and the distribution of the nodules in the ACR TI-RADS category in the retrospective set and the prospective set are shown in eTable 2 in Supplement 1. Details for other image features are presented in eTable 3 in Supplement 1.

### Identifying Nonsignificant and Significant Features for Optimized Strategy

#### Nonsignificant Features

**Figure 2.**
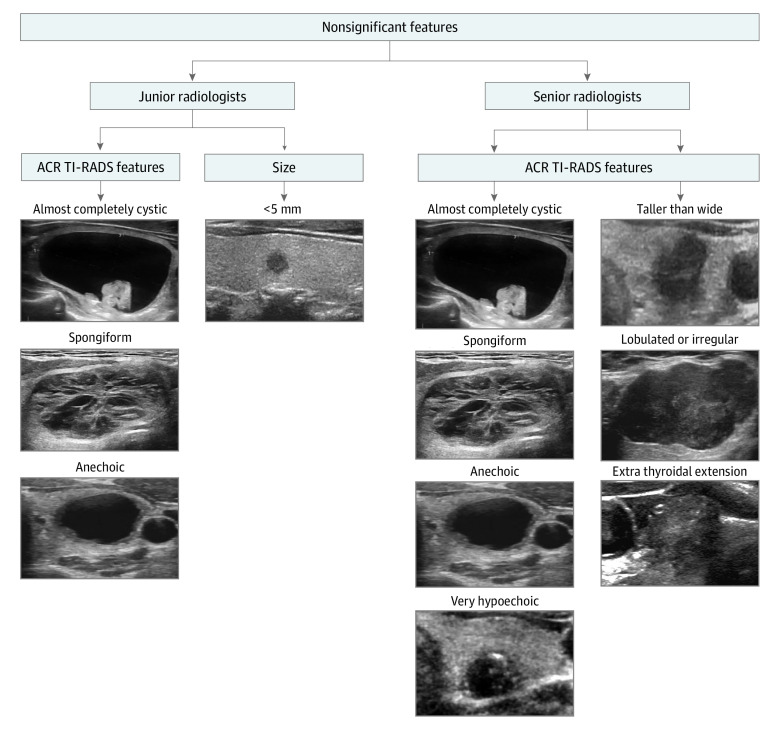
Nonsignificant Features for Junior and Senior Radiologists The nonsignificant features for junior radiologists included cystic or almost completely cystic nodules, spongiform nodules, anechoic nodules, and nodule size less than 5 mm, whereas nonsignificant features for senior radiologists were cystic or almost completely cystic nodules, spongiform nodules, anechoic nodules, very hypoechoic nodules, nodules taller than wide, lobulated or irregular nodules, and extrathyroidal extension. ACR TI-RADS indicates American College of Radiology Thyroid Imaging Reporting and Data System.

As shown in Figure 2 and eTables 4 to 10 in the Supplement, for junior radiologists, we investigated the following nonsignificant ultrasonographic features that were not statistically significantly improved by AI assistance: cystic or almost completely cystic nodules, anechoic nodules, spongiform nodules, and nodules smaller than 5 mm. For senior radiologists, the nonsignificant features included cystic or almost completely cystic nodules, anechoic nodules, spongiform nodules, very hypoechoic nodules, nodules taller than wide, lobulated or irregular nodules, and extrathyroidal extension. Among junior radiologists, the artificial intelligence assistance showed a slight decrease in the specificity of cystic or almost completely cystic nodules (from 98% [95% CI, 96%-100%] to 97% [95% CI, 95%-99%]; *P* = .55), anechoic nodules (from 98% [95% CI, 96%-100%] to 97% [95% CI, 95%-99%]; *P* = .55), and nodule size less than 5 mm (from 68% [95% CI, 58%-78%] to 67% [95% CI, 57%-77%]; *P* > .99).

#### Significant Features

Our study revealed that use of AI assistance was associated with significant improvements in sensitivity and/or specificity across various ultrasonographic features (eTables 4-10 and eFigures 1 and 2 in Supplement 1). For junior radiologists, significant features included mixed cystic and solid nodules, solid or almost completely solid nodules, hyperechoic or isoechoic nodules, hypoechoic nodules, very hypoechoic nodules, all shapes, all margin features, none or large comet-tail artifacts, macrocalcifications and punctate echogenic foci, sizes of 5 mm or more, and all parenchymal backgrounds. Improvements ranged from 44% (95% CI, 36%-52%) to 99% (95% CI, 97%-100%) for sensitivity (all *P* ≤ .004 except for mixed cystic and solid nodules with *P* > .05) and from 33% (95% CI, 28%-38%) to 96% (95% CI, 95%-97%) for specificity (all *P* < .001 except for very hypoechoic nodules, nodules wider than tall, smooth nodules, lobulated or irregular nodules, and extrathyroidal extension with *P* > .05). For senior radiologists, significant features included mixed cystic and solid nodules, solid or almost completely solid nodules, hyperechoic or isoechoic nodules, hypoechoic nodules, nodules wider than tall, smooth nodules, ill-defined nodules, none or large comet-tail artifacts, macrocalcifications, punctate echogenic foci, all sizes, and all parenchymal backgrounds. Improvements ranged from 43% (95% CI, 35%-52%) to 97% (95% CI, 96%-98%) for sensitivity (all *P* ≤ .009 except for mixed cystic and solid nodules, nodules smaller than 5 mm with *P* > .05) and from 38% (95% CI, 33%-43%) to 97% (95% CI, 96%-98%) for specificity (all *P* ≤ .04 except for macrocalcifications and nodules range from 5 to 10 mm with *P* > .05).

### Testing Optimized Strategy in Prospective Set

The prospective study involved conducting a traditional full AI strategy diagnosis and an optimized strategy diagnosis, comparing the diagnostic performance of these 2 strategies for both junior and senior radiologists, and analyzing the time-based cost difference for each reader when using these 2 strategies to diagnose images with nonsignificant features.

#### Diagnostic Performance

In the overall comparison, we did not observe any significant differences in diagnostic sensitivity and specificity between the traditional all-AI strategy and the optimized strategy diagnosis among all radiologists in our study. The sensitivity ranged from 91% to 100%, and the specificity ranged from 94% to 98% for readers 11 to 16 (Table 1).

**Table 1.** Comparison of Traditional All-AI Strategy and Optimized Strategy in Sensitivity and Specificity for Each Radiologist

Radiologist	Sensitivity (95% CI), %			Specificity (95% CI), %		
	Traditional all-AI strategy	Optimized strategy	*P* value	Traditional all-AI strategy	Optimized strategy	*P* value
Junior radiologists						
Reader 11	100	100	>.99	98 (95-100)	96 (93-100)	.50
Reader 12	97 (94-99)	97 (95-100)	>.99	95 (91-99)	94 (90-99)	>.99
Reader 13	91 (87-96)	91 (87-95)	>.99	94 (89-98)	94 (90-98)	>.99
Senior radiologists						
Reader 14	98 (96-100)	98 (96-100)	>.99	96 (93-100)	94 (90-99)	.50
Reader 15	97 (95-99)	95 (92-98)	.13	95 (91-99)	94 (90-98)	>.99
Reader 16	95 (92-99)	95 (92-98)	>.99	97 (94-100)	94 (89-98)	.13

Abbreviation: AI, artificial intelligence.

#### Time-Based Cost

**Figure 3.**
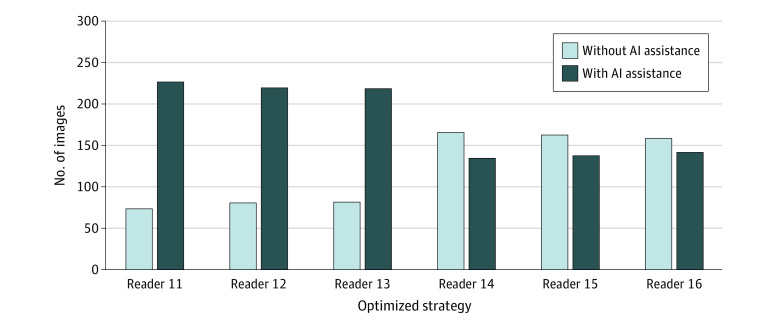
Identification of Nonsignificant Features in 300 Images by 6 Radiologists In the set of 300 images, junior radiologist readers 11 to 13 identified 73 (24.3%), 80 (26.7%), and 81 (27.0%) images, respectively, with nonsignificant features. Senior radiologist readers 14 to 16 identified 166 (55.3%), 162 (54.0%), and 158 (52.7%) images, respectively, with nonsignificant features.

In the prospective set of 300 images, junior radiologist readers 11 to 13 identified 73 (24.3%), 80 (26.7%), and 81 (27.0%) images, respectively, with nonsignificant features, while senior radiologist readers 14 to 16 identified 166 (55.3%), 162 (54.0%), and 158 (52.7%) images, respectively, with nonsignificant features (Figure 3). After implementing the optimized strategy, the total time-based cost for junior radiologists increased from 15 125 seconds to 15 623 seconds, while the total time-based cost for senior radiologists decreased from 12 721 seconds to 11 892 seconds. The difference in time-based cost at the point of divergence between the 2 strategies manifested mainly in the diagnosis of images with nonsignificant features. As shown in Table 2, in these readings of images with nonsignificant features, junior radiologist reader 11 and reader 12 did not save time but instead increased their total time by 311 seconds and 239 seconds, respectively. The mean time-based cost increased from 15.2 seconds (95% CI, 13.2-17.2 seconds) to 19.4 seconds (95% CI, 15.6-23.3 seconds) for reader 11 and increased from 12.7 seconds (95% CI, 11.4-13.9 seconds) to 15.6 seconds (95% CI, 13.6-17.7 seconds) for reader 12, with a significant increase in time-based cost per case of 4.3 seconds (95% CI, −7.7 to −0.8 seconds; *P* = .02) for reader 11 and 3.0 seconds (95% CI, −4.7 to −1.3 seconds; *P* = .001) for reader 12. In contrast, senior radiologist readers 14 to 16 saved time in total after using the optimized strategy, with decreases of 432 seconds, 406 seconds, and 86 seconds, respectively. Among them, reader 14 and reader 15 had significant reductions in the mean time-based cost, from 19.4 seconds (95% CI, 18.1-20.7 seconds) to 16.8 seconds (95% CI, 15.3-18.3 seconds) for reader 14 and from 12.5 seconds (95% CI, 12.1-12.9 seconds) to 10.0 seconds (95% CI, 9.5-10.5 seconds) for reader 15, with each case having a significant decrease in the time-based cost of 2.6 seconds (95% CI, 1.0-4.2 seconds; *P* = .001) for reader 14 and 2.5 seconds (95% CI, 2.0-3.1 seconds; *P* < .001) for reader 15.

**Table 2.** Comparison of Traditional All-AI Strategy and Optimized Strategy in Time-Based Cost of Nonsignificant Feature Images

Radiologist	Total cost of traditional strategy, s	Total cost of optimized strategy, s	Total cost saving, s	Mean cost of traditional strategy, 95% CI, s	Mean cost of optimized strategy, 95% CI, s	Mean cost saving, 95% CI, s	*P* value
Junior radiologists							
Reader 11 (n = 73)^a^	1108	1419	−311	15.2 (13.2 to 17.2)	19.4 (15.6 to 23.3)	−4.3 (−7.7 to −0.8)	.02
Reader 12 (n = 80)^a^	1012	1251	−239	12.7 (11.4 to 13.9)	15.6 (13.6 to 17.7)	−3.0 (−4.7 to −1.3)	.001
Reader 13 (n = 81)^a^	965	940	25	11.9 (10.0 to 13.9)	11.6 (10.3 to 12.8)	0.3 (−2.0 to 2.6)	.79
Senior radiologists							
Reader 14 (n = 166)^a^	3221	2789	432	19.4 (18.1 to 20.7)	16.8 (15.3 to 18.3)	2.6 (1.0 to 4.2)	.001
Reader 15 (n = 162)^a^	2027	1621	406	12.5 (12.1 to 12.9)	10.0 (9.5 to 10.5)	2.5 (2.0 to 3.1)	<.001
Reader 16 (n = 158)^a^	1496	1410	86	9.5 (8.9 to 10.1)	8.9 (8.4 to 9.4)	0.5 (−0.02 to 1.1)	.06

Abbreviation: AI, artificial intelligence.

^a^ The number of images with nonsignificant features.

## Discussion

Effective human-AI integration is necessary for thyroid nodule management to provide real clinical benefit. In this study, we explored for the first time, to our knowledge, an approach to build an integraded AI. By evaluating the association between ultrasonographic image features and AI-assisted diagnostic performance, we developed an optimized strategy to inform how radiologists with different expertise levels could benefit when integrating AI-generated recommendations. The feasibility of this strategy was then tested according to time-based cost and diagnostic performance to assess the preferable mode of human-AI interaction. We found that the optimized strategy was able to maintain excellent diagnostic performance for both junior and senior radiologists compared with the traditional all-AI strategy. In terms of reducing the time-based cost, the traditional all-AI strategy was preferable for junior radiologists, whereas the optimized strategy was better suited for senior radiologists.

Currently, a lack of trust in AI systems is a significant impediment to the adoption of this technology in medical diagnosis.^14,15^ Building optimized mechanisms requires an understanding of suitable AI-assisted scenarios in which radiologists can fully trust AI-generated recommendations. The adoption of AI can be influenced by several factors, such as the properties of the AI system, user experiences, and the design of the human-AI collaboration. In previous studies, some features, such as sonographic signs, the contrast of the image, and nodule size, had implications for the AI-generated diagnosis.^16,17,18^ As a result, AI technology may not have performed a task predictably and consistently; thus, reliability would be affected. Our results showed that, for junior radiologists, the ultrasonographic features that were not improved by AI assistance were typical benign features, such as cystic or almost completely cystic, anechoic, and spongiform nodules.^19,20^ In addition to these factors, for senior radiologists, the ultrasonographic features that were not improved by AI assistance were typical malignant features, such as very hypoechoic nodules, nodules taller than wide, lobulated or irregular nodules, and extrathyroidal extension nodules. We considered that radiologists possess a high level of diagnostic performance and confidence with regard to these typical nodules, allowing them to be confident in their diagnosis without AI support when this sole feature appeared, which explained why, as long as the nonsignificant feature was present, it could be evaluated independent of AI support in our study. Therefore, we proposed a human-AI interaction strategy to integrate the radiologists’ experience to match the knowledge learned by the AI model. This more cautious approach to human-AI interaction will allow for safer clinical deployment and should engender trust with the radiologists who use this technology. This technology will help radiologists to feel confident in the diagnostic information the model has provided and to evaluate when this information should be questioned.

Our study found a difference in the time-based cost between junior and senior radiologists in the prospective set simulating the optimized strategy. Compared with the traditional all-AI strategy, the time-based cost of the optimized strategy was higher for junior radiologists and lower for senior radiologists. This difference demonstrates that AI can both improve the accuracy of diagnosis and pass false information to radiologists. For junior radiologists, AI is of great help to improve diagnosis and can speed up the decision-making workflow. For senior radiologists, however, false information from AI can hinder decision-making. Our optimized strategy, which includes independent diagnosis, reduced the chance of these conflicting judgments, thereby reducing the time-based cost. Thus, developing relevant application specifications to guide AI in clinical practice is necessary. As our results show, the traditional all-AI strategy was preferable for junior radiologists, whereas the optimized strategy was better suited for senior radiologists. These results reveal a promising approach to implementing AI-assisted precision medicine.

### Limitations

This study has several limitations. First, a more rigorous study design would have included a crossover design to reduce possible recall bias. However, we implemented a washout period of 4 weeks and ensured that the radiologists were not provided with pathology results during the previous reading process, which may have alleviated recall bias to some extent.^21,22^ Second, we did not explore deep learning interpretability in algorithms because model interpretability is important for the adoption of AI-based health care applications. Third, other factors (such as personality or risky decision-making ability) may also affect the performance of AI assistance, and such factors should be explored in future studies.^23^ Fourth, because the study was conducted in a simulated clinical environment, the actual implementation of human-AI collaboration needs to be confirmed in future research.^24^

## Conclusions

On the basis of the findings in this diagnostic study, we recommend that junior radiologists apply the traditional all-AI strategy in thyroid nodule management, whereas senior radiologists should apply the optimized strategy. These optimized integrations of AI decision aids have the potential to help radiologists reduce workload by decreasing diagnostic time-based costs while maintaining excellent diagnostic performance.

## Notes

Supplement 1. **eTable 1.** Basic Information of all Radiologists
eMethods.

eReference.
**eTable 2.** The Demographic Characteristics of Patients and the Nodules Distribution of ACR TI-RADS Category **eTable 3.** Distribution of Images Features **eTable 4.** Comparison of Independent Diagnosis and AI Model Assistance in Sensitivity and Specificity Under Different ACR TI-RADS Composition Features in Junior and Senior Radiologists **eTable 5.** Comparison of Independent Diagnosis and AI Model Assistance in Sensitivity and Specificity Under Different ACR TI-RADS Echogenicity Features in Junior and Senior Radiologists **eTable 6.** Comparison of Independent Diagnosis and AI Model Assistance in Sensitivity and Specificity Under Different ACR TI-RADS Shape Features in Junior and Senior Radiologists **eTable 7.** Comparison of Independent Diagnosis and AI Model Assistance in Sensitivity and Specificity Under Different ACR TI-RADS Margin Features in Junior and Senior Radiologists **eTable 8.** Comparison of Independent Diagnosis and AI Model Assistance in Sensitivity and Specificity Under Different ACR TI-RADS Echogenic Foci Features in Junior and Senior Radiologists **eTable 9.** Comparison of Independent Diagnosis and AI Model Assistance in Sensitivity and Specificity Under Different Nodule Sizes in Junior and Senior Radiologists **eTable 10.** Comparison of Independent Diagnosis and AI Model Assistance in Sensitivity and Specificity Under Different Parenchymal Backgrounds in Junior and Senior Radiologists **eFigure 1.** The Significant Features for Junior Radiologists **eFigure 2.** The Significant Features for Senior Radiologists

Supplement 2.
Data Sharing Statement
