# Supplementary material for: Integration of Artificial Intelligence Decision Aids to Reduce Workload and Enhance Efficiency in Thyroid Nodule Management
Source: JAMA Netw Open. 2023 May 16;6(5):e2313674. doi: 10.1001/jamanetworkopen.2023.13674 (PMC10189570; doi:10.1001/jamanetworkopen.2023.13674)
Supplement: Supplement 1. — eTable 1. Basic Information of all Radiologists eMethods. eReference. eTable 2. The Demographic Characteristics of Patients and the Nodules Distribution of ACR TI-RADS Category eTable 3. Distribution of Images Features eTable 4. Comparison of Independent Diagnosis and AI Model Assistance in Sensitivity and Specificity Under Different ACR TI-RADS Composition Features in Junior and Senior Radiologists eTable 5. Comparison of Independent Diagnosis and AI Model Assistance in Sensitivity and Specificity Under Different ACR TI-RADS Echogenicity Features in Junior and Senior Radiologists eTable 6. Comparison of Independent Diagnosis and AI Model Assistance in Sensitivity and Specificity Under Different ACR TI-RADS Shape Features in Junior and Senior Radiologists eTable 7. Comparison of Independent Diagnosis and AI Model Assistance in Sensitivity and Specificity Under Different ACR TI-RADS Margin Features in Junior and Senior Radiologists eTable 8. Comparison of Independent Diagnosis and AI Model Assistance in Sensitivity and Specificity Under Different ACR TI-RADS Echogenic Foci Features in Junior and Senior Radiologists eTable 9. Comparison of Independent Diagnosis and AI Model Assistance in Sensitivity and Specificity Under Different Nodule Sizes in Junior and Senior Radiologists eTable 10. Comparison of Independent Diagnosis and AI Model Assistance in Sensitivity and Specificity Under Different Parenchymal Backgrounds in Junior and Senior Radiologists eFigure 1. The Significant Features for Junior Radiologists eFigure 2. The Significant Features for Senior Radiologists [file jamanetwopen-e2313674-s001.pdf]

Supplementary Online Content

Tong WJ, Wu SH, Cheng MQ, et al. Integration of artificial intelligence decision aids to reduce workload and enhance efficiency in thyroid nodule management. *JAMA Netw Open.* 2023;6(5):e2313674. doi:10.1001/jamanetworkopen.2023.13674

eTable 1. Basic Information of all Radiologists
eMethods.
eReference.
eTable 2. The Demographic Characteristics of Patients and the Nodules Distribution of ACR TI-RADS Category
eTable 3. Distribution of Images Features
eTable 4. Comparison of Independent Diagnosis and AI Model Assistance in Sensitivity and Specificity Under Different ACR TI-RADS Composition Features in Junior and Senior Radiologists
eTable 5. Comparison of Independent Diagnosis and AI Model Assistance in Sensitivity and Specificity Under Different ACR TI-RADS Echogenicity Features in Junior and Senior Radiologists
eTable 6. Comparison of Independent Diagnosis and AI Model Assistance in Sensitivity and Specificity Under Different ACR TI-RADS Shape Features in Junior and Senior Radiologists
eTable 7. Comparison of Independent Diagnosis and AI Model Assistance in Sensitivity and Specificity Under Different ACR TI-RADS Margin Features in Junior and Senior Radiologists
eTable 8. Comparison of Independent Diagnosis and AI Model Assistance in Sensitivity and Specificity Under Different ACR TI-RADS Echogenic Foci Features in Junior and Senior Radiologists
eTable 9. Comparison of Independent Diagnosis and AI Model Assistance in Sensitivity and Specificity Under Different Nodule Sizes in Junior and Senior Radiologists
eTable 10. Comparison of Independent Diagnosis and AI Model Assistance in Sensitivity and Specificity Under Different Parenchymal Backgrounds in Junior and Senior Radiologists
eFigure 1. The Significant Features for Junior Radiologists
eFigure 2. The Significant Features for Senior Radiologists

This supplementary material has been provided by the authors to give readers additional information about their work.

© 2023 Tong WJ et al. *JAMA Network Open.*

eTable 1. Basic Information of all Radiologists

	Radiologist		years	Work Seniority (as radiologists, year)	Degree	Average examinations performed per month
**Retrospective set**	Junior radiologists	Reader 1	29	1.5	PhD	900
		Reader 2	25	1.5	MM	800
		Reader 3	34	2	MM	1200
		Reader 4	31	2	PhD	800
		Reader 5	32	3	MD	600
	Senior radiologists	Reader 6	37	8	MM	780
		Reader 7	39	9	PhD	800
		Reader 8	34	8	BMS	850
		Reader 9	38	10	PhD	1100
		Reader 10	37	12	MM	850
**Prospective set**	Junior radiologists	Reader 11	29	1.5	MD	650
		Reader 12	29	1.5	MM	780
		Reader 13	30	2	MM	800
	Senior radiologists	Reader 14	37	10	MM	830
		Reader 15	33	6	MM	1050
		Reader 16	37	10	PhD	1180

Abbreviations: PhD, Doctor of Philosophy; MM, Master of Medicine; MD, Doctor of Medicine; BMS, Bachelor of Medical Science.

© 2023 Tong WJ et al. *JAMA Network Open.*

eMethods.

(1) The definitions of junior and senior radiologists are as follows:

Junior radiologists are doctors who have less than 3 years of experience in performing ultrasound scans. They undergo a 3-year standardized national resident training program, which includes standardized instruction in conducting thyroid ultrasound examinations. On average, they evaluate thyroid ultrasound images from approximately 600 patients per year. Senior radiologists, on the other hand, have more than 5 years of experience in thyroid ultrasound and have completed both the standardized national resident training and specialist training in ultrasound. They review thyroid ultrasound images from an average of about 800 patients per year.

(2) AI model establishment and evaluation

The deep-learning AI model was established in previous study[1]. For the training set, a total of 18049 ultrasound images from 8339 patients with thyroid nodules were retrospectively retrieved from the individual thyroid imaging database at the First Affiliated Hospital of Sun Yat-sen University and Sun Yat-sen University Cancer Center, Guangzhou, China. AI deep-learning algorithm was specifically designed to diagnose malignancy from benign thyroid nodules, with a combined architecture of three networks: ResNet, ResNeXt, and DenseNet. To search for the optimal weights for each network branch and get the ensembled output, we used the brute-force search method via cross-test in the training sets. The final weighting ratios are 0.40 for ResNet, 0.35 for ResNeXt, and 0.25 for DenseNet. Then three independent testing sets were used to evaluate the AI model. In test A, the diagnostic performance of stand-alone AI was compared with radiologists. The results showed that AI achieved significantly higher accurate diagnosis (AUROC: 0.922, [95% CI 0.910-0.934]) than radiologists (AUROC: 0.839, [95% CI 0.834-0.844]; P < .0001). In test B, improvement in the diagnostic performance of -AI-assisted strategy was evaluated. AI assistance improved the pooled AUROC of the radiologists from 0.855 (95% CI 0.848-0.862) when diagnosing without AI to 0.885 (95% CI 0.879-0.891; P < .0001) for the senior radiologists, and from 0.819 (95% CI 0.811-0.826) to 0.866 (95%CI 0.859-0.872; P < .0001) for the junior radiologist. In the simulated scenario of clinical reading using images and videos in test C, the pooled AUROC of the final diagnosis with AI assistance was improved to 0.873 (95% CI 0.863-0.83) from the 0.862 (95% CI 0.851-0.872; p < .0001) of radiologists reviewing videos and images without AI assistance. The details of training and validation for AI model establishment can be found in previous article[1]. The AI model is available via the web page http://119.91.96.49:8686/ (established by Maiying Technology Co., Ltd, Guangzhou, China).

eReference.

1. Peng S, Liu Y, Lv W, et al. Deep learning-based artificial intelligence model to assist thyroid nodule diagnosis and management: a multicentre diagnostic study. *The Lancet Digital health*. 2021;3(4):e250-e259. doi:10.1016/s2589-7500(21)00041-8

© 2023 Tong WJ et al. *JAMA Network Open.*

eTable 2. The Demographic Characteristics of Patients and the Nodules Distribution of ACR TI-RADS Category

		Retrospective set (n=1754)	Prospective set (n=300)
**Sex**	Male	439 (25.0)	82 (27.3)
	Female	1315 (75.0)	218 (72.7)
**Age (years)**	Mean (SD)	42.1 (13.2)	41.7 (14.1)
	Median (IQR)	41 (33-52)	40 (32-53)
**ACR TI-RADS category**	TR 1	95 (5.4)	54 (18.0)
	TR 2	112 (6.4)	48 (16.0)
	TR 3	229 (13.1)	6 (2.0)
	TR 4	459 (26.2)	34 (11.3)
	TR 5	859 (49.0)	158 (52.7)

Abbreviations: ACR TI-RADS, The American College of Radiology published the Thyroid Imaging Reporting and Data System; IQR, inter-quartile range; SD, standard deviation; TR, TI-RADS category.

Sex and ACR TI-RADS category are listed as numbers of images, while age is listed as the number of patients.

Except where indicated, data in parentheses are percentages.

© 2023 Tong WJ et al. *JAMA Network Open.*

eTable 3. Distribution of Images Features

Variable	Retrospective set (n=1754)		Prospective set (n=300)	
	Benign (n=748)	Malignant (n=1006)	Benign (n=125)	Malignant (n=175)
**ACR TI-RADS features**				
**Composition**				
Cystic or almost completely cystic	60 (8.0)	0 (0.0)	30 (24.0)	0 (0.0)
Spongiform	35 (4.7)	0 (0.0)	24 (19.2)	0 (0.0)
Mixed cystic and solid	167 (22.6)	6 (0.6)	57 (45.6)	5 (2.9)
Solid or almost completely solid	486 (65.0)	1000 (94.0)	14 (11.2)	170 (97.1)
**Echogenicity**				
Anechoic	60 (8.0)	0 (0.0)	30 (24.0)	0 (0.0)
Hyperechoic or isoechoic	354 (47.3)	29 (2.7)	65 (52.0)	6 (3.4)
Hypoechoic	327 (43.7)	792 (74.4)	24 (19.2)	117 (66.9)
Very hypoechoic	7 (0.9)	185 (17.4)	6 (4.8)	52 (29.7)
**Shape**				
Wider-than-tall	712 (95.2)	594 (55.8)	123 (98.4)	99 (56.6)
Taller-than-wide	36 (4.8)	412 (38.7)	2 (1.6)	76 (43.4)
**Margin**				
Smooth	333 (44.5)	45 (4.2)	94 (75.2)	8 (4.6)
Ill-defined	379 (50.7)	587 (55.2)	28 (22.4)	76 (43.4)
Lobulated or irregular	35 (4.7)	340 (32.0)	3 (2.4)	73 (41.7)
Extra-thyroidal extension	1 (0.1)	34 (3.2)	0 (0.0)	18 (10.3)
**Echogenic foci**				
None or large comet-tail artifacts	619 (82.8)	394 (37.0)	110 (88.0)	46 (26.3)
Macrocalcifications	62 (8.3)	144 (13.5)	9 (7.2)	42 (24.0)
Peripheral (rim) calcifications	13 (1.7)	13 (1.2)	2 (1.6)	3 (1.7)
Punctate echogenic foci	70 (9.4)	522 (49.1)	9 (7.2)	107 (61.1)
**Nodule size**				
< 5 mm	18 (2.4)	63 (5.9)	6 (4.8)	13 (7.4)
5 mm-10 mm	94 (12.6)	400 (37.6)	11 (8.8)	75 (42.9)
10 mm-20 mm	242 (32.4)	427 (40.1)	30 (24.0)	61 (34.9)
> 20 mm	394 (52.7)	116 (10.9)	78 (62.4)	26 (14.9)
**Parenchyma**				
Normal parenchyma	507 (67.8)	739 (69.5)	82 (65.6)	103 (58.9)
Diffused parenchyma	241 (32.2)	267 (25.1)	43 (34.4)	72 (41.1)

Abbreviations: ACR TI-RADS, The American College of Radiology published the Thyroid Imaging Reporting and Data

© 2023 Tong WJ et al. *JAMA Network Open.*

System. Data are number of nodules, with percentages in parentheses.

eTable 4. Comparison of Independent Diagnosis and AI Model Assistance in Sensitivity and Specificity Under Different ACR TI-RADS Composition Features in Junior and Senior Radiologists

	Sensitivity		p value	Specificity		p value
	without AI (%)	with AI (%)		without AI (%)	with AI (%)	
**Cystic or almost completely cystic**						
Junior				98 (96, 100)	97 (95, 99)	.55
Senior				99 (97, 100)	99 (97, 100)	>.99
**Spongiform**						
Junior				91 (87, 95)	93 (89, 97)	.39
Senior				93 (89, 97)	94 (91, 98)	.73
**Mixed cystic and solid**						
Junior	37 (18, 55)	40 (21, 59)	>.99	92 (91, 94)	96 (95, 97)	<.001
Senior	57 (38, 75)	63 (45, 82)	.63	95 (94, 97)	97 (96, 98)	.001
**Solid or almost completely solid**						
Junior	89 (88, 89)	93 (92, 93)	<.001	67 (65, 69)	73 (71, 75)	<.001
Senior	91 (90, 91)	93 (92, 94)	<.001	72 (70, 74)	75 (74, 77)	<.001

Abbreviations: ACR TI-RADS, The American College of Radiology published the Thyroid Imaging Reporting and Data System; AI, Artificial intelligence.

Numbers in parentheses are 95% CIs.

© 2023 Tong WJ et al. *JAMA Network Open.*

eTable 5. Comparison of Independent Diagnosis and AI Model Assistance in Sensitivity and Specificity Under Different ACR TI-RADS Echogenicity Features in Junior and Senior Radiologists

	**Sensitivity**		**p value**	**Specificity**		**p value**
	**without AI (%)**	**with AI (%)**		**without AI (%)**	**with AI (%)**	
**Anechoic**						
Junior				98 (96, 100)	97 (95, 99)	.55
Senior				99 (97, 100)	99 (97, 100)	>.99
**Hyperechoic or isoechoic**						
Junior	44 (36, 52)	55 (47, 63)	.001	90 (89, 92)	94 (93, 95)	<.001
Senior	43 (35, 52)	57 (49, 65)	.001	94 (93, 95)	96 (95, 97)	.002
**Hypoechoic**						
Junior	89 (88, 90)	93 (92, 94)	<.001	59 (56, 61)	65 (63, 67)	<.001
Senior	91 (90, 92)	93 (93, 94)	<.001	64 (61, 66)	68 (65, 70)	<.001
**Very hypoechoic**						
Junior	93 (92, 95)	97 (96, 98)	<.001	17 (4, 30)	23 (8, 37)	.75
Senior	96 (95, 97)	96 (95, 98)	.68	14 (2, 26)	34 (18, 51)	.07

Abbreviations: ACR TI-RADS, The American College of Radiology published the Thyroid Imaging Reporting and Data System; AI, Artificial intelligence.

Numbers in parentheses are 95% CIs.

© 2023 Tong WJ et al. *JAMA Network Open.*

eTable 6. Comparison of Independent Diagnosis and AI Model Assistance in Sensitivity and Specificity Under Different ACR TI-RADS Shape Features in Junior and Senior Radiologists

	Sensitivity		p value	Specificity		p value
	without AI (%)	with AI (%)		without AI (%)	with AI (%)	
**Wider-than-tall**						
Junior	84 (82, 85)	89 (88, 90)	<.001	79 (78, 80)	84 (82, 85)	<.001
Senior	86 (85, 87)	90 (89, 90)	<.001	83 (82, 84)	86 (84, 87)	<.001
**Taller-than-wide**						
Junior	95 (94, 96)	97 (96, 98)	<.001	24 (18, 31)	28 (21, 34)	.29
Senior	96 (95, 97)	97 (96, 98)	.08	31 (24, 37)	33 (26, 40)	.36

Abbreviations: ACR TI-RADS, The American College of Radiology published the Thyroid Imaging Reporting and Data System; AI, Artificial intelligence.

Numbers in parentheses are 95% CIs.

© 2023 Tong WJ et al. *JAMA Network Open.*

eTable 7. Comparison of Independent Diagnosis and AI Model Assistance in Sensitivity and Specificity Under Different ACR TI-RADS Margin Features in Junior and Senior Radiologists

	Sensitivity		p value	Specificity		p value
	without AI (%)	with AI (%)		without AI (%)	with AI (%)	
**Smooth**						
Junior	45 (38, 51)	60 (53, 66)	<.001	93 (91, 94)	94 (93, 95)	.08
Senior	46 (40, 53)	57 (51, 64)	<.001	95 (94, 96)	96 (95, 97)	.033
**Ill-defined**						
Junior	87 (86, 88)	92 (91, 93)	<.001	68 (66, 70)	75 (73, 77)	<.001
Senior	90 (89, 91)	93 (92, 94)	<.001	74 (72, 76)	78 (76, 79)	<.001
**Lobulated or irregular**						
Junior	95 (94, 96)	97 (96, 97)	.001	14 (9, 20)	18 (13, 24)	.14
Senior	97 (96, 97)	97 (96, 98)	.65	19 (13, 25)	22 (16, 28)	.41
**Extra-thyroid al extension**						
Junior	95 (91, 98)	99 (97, 100)	.004	0	60 (-8, 128)	.25
Senior	98 (95, 100)	98 (96, 100)	>.99	0	40 (-3, 108)	.50

Abbreviations: ACR TI-RADS, The American College of Radiology published the Thyroid Imaging Reporting and Data System; AI, Artificial intelligence.

Numbers in parentheses are 95% CIs.

© 2023 Tong WJ et al. *JAMA Network Open.*

eTable 8. Comparison of Independent Diagnosis and AI Model Assistance in Sensitivity and Specificity Under Different ACR TI-RADS Echogenic Foci Features in Junior and Senior Radiologists

	Sensitivity		p value	Specificity		p value
	without AI (%)	with AI (%)		without AI (%)	with AI (%)	
**None or large comet-tail artifacts**						
Junior	80 (78, 82)	87 (86, 89)	<.001	84 (83, 86)	87 (86, 88)	<.001
Senior	85 (83, 87)	87 (86, 89)	<.001	87 (86, 89)	90 (88, 91)	<.001
**Macrocalcifications**						
Junior	91 (89, 93)	95 (93, 96)	<.001	42 (36, 47)	52 (46, 57)	<.001
Senior	91 (89, 93)	95 (94, 97)	<.001	54 (48, 59)	56 (51, 62)	.32
**Peripheral (rim) calcifications**						
Junior	69 (58, 81)	77 (66, 87)	.13	57 (45, 69)	60 (48, 72)	.77
Senior	60 (48, 72)	79 (68, 89)	.004	68 (56, 79)	60 (48, 72)	.23
**Punctate echogenic foci**						
Junior	95 (94, 95)	97 (96, 97)	<.001	33 (28, 38)	46 (40, 51)	<.001
Senior	95 (94, 96)	97 (96, 98)	<.001	38 (33, 43)	45 (40, 50)	<.001

Abbreviations: ACR TI-RADS, The American College of Radiology published the Thyroid Imaging Reporting and Data System; AI, Artificial intelligence.

Numbers in parentheses are 95% CIs.

© 2023 Tong WJ et al. *JAMA Network Open.*

eTable 9. Comparison of Independent Diagnosis and AI Model Assistance in Sensitivity and Specificity Under Different Nodule Sizes in Junior and Senior Radiologists

	Sensitivity		p value	Specificity		p value
	**without AI (%)**	**with AI (%)**		**without AI (%)**	**with AI (%)**	
**< 5 mm**						
Junior	86 (82, 90)	89 (85, 92)	.14	68 (58, 78)	67 (57, 77)	>.99
Senior	89 (86, 93)	92 (89, 95)	.11	64 (54, 75)	73 (64, 83)	.039
**5 mm-10 mm**						
Junior	88 (87, 90)	93 (92, 94)	<.001	57 (53, 62)	64 (59, 68)	<.001
Senior	91 (90, 93)	93 (92, 94)	.001	64 (60, 68)	66 (61, 70)	.33
**10 mm-20 mm**						
Junior	90 (89, 91)	95 (94, 95)	<.001	68 (65, 71)	74 (71, 76)	<.001
Senior	92 (91, 93)	95 (94, 96)	<.001	73 (70, 75)	76 (74, 78)	<.001
**> 20 mm**						
Junior	82 (79, 85)	86 (83, 89)	<.001	86 (85, 88)	90 (89, 91)	<.001
Senior	81 (78, 84)	84 (81, 87)	.009	90 (89, 91)	92 (91. 93)	<.001

Abbreviations: AI, Artificial intelligence.

Numbers in parentheses are 95% CIs.

© 2023 Tong WJ et al. *JAMA Network Open.*

eTable 10. Comparison of Independent Diagnosis and AI Model Assistance in Sensitivity and Specificity Under Different Parenchymal Backgrounds in Junior and Senior Radiologists

	Sensitivity		p value	Specificity		p value
	without AI (%)	with AI (%)		without AI (%)	with AI (%)	
**Homogeneous parenchyma**						
Junior	88 (87, 89)	92 (91, 93)	<.001	76 (74, 77)	80 (78, 81)	<.001
Senior	90 (89, 91)	93 (92. 93)	<.001	79 (78, 81)	82 (81, 84)	<.001
**Heterogeneous parenchyma**						
Junior	90 (88, 91)	94 (93, 95)	<.001	77 (75, 80)	83 (81, 85)	<.001
Senior	90 (89, 92)	94 (93, 95)	<.001	83 (81, 85)	85 (83, 87)	.028

Abbreviations: AI, Artificial intelligence.

Numbers in parentheses are 95% CIs.

© 2023 Tong WJ et al. *JAMA Network Open.*

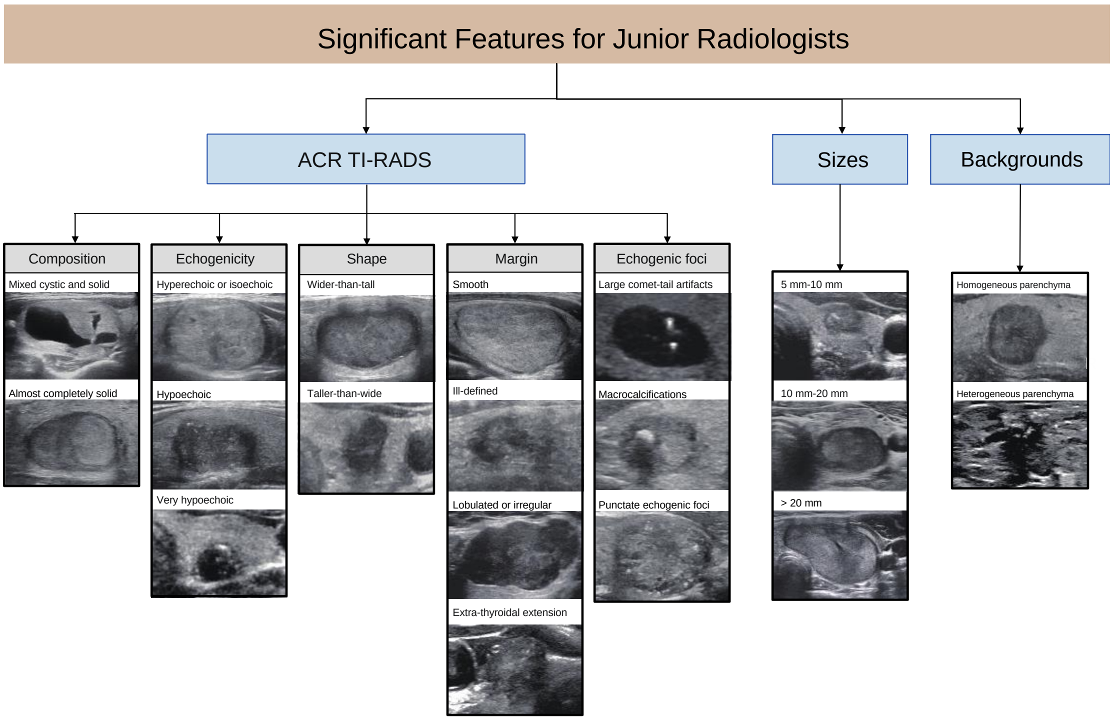

eFigure 1. The Significant Features for Junior Radiologists

For junior radiologists, mixed cystic and solid, solid or almost completely solid, hyperechoic or isoechoic, hypoechoic, very hypoechoic, wider-than-tall, taller-than-wide, smooth, ill-defined, lobulated or irregular, extra-thyroidal extension, none or large comet-tail artifacts, macrocalcifications, punctate echogenic foci, homogeneous and heterogeneous parenchyma, and nodule sizes ≥ 5mm were investigated as significant features.

© 2023 Tong WJ et al. *JAMA Network Open.*

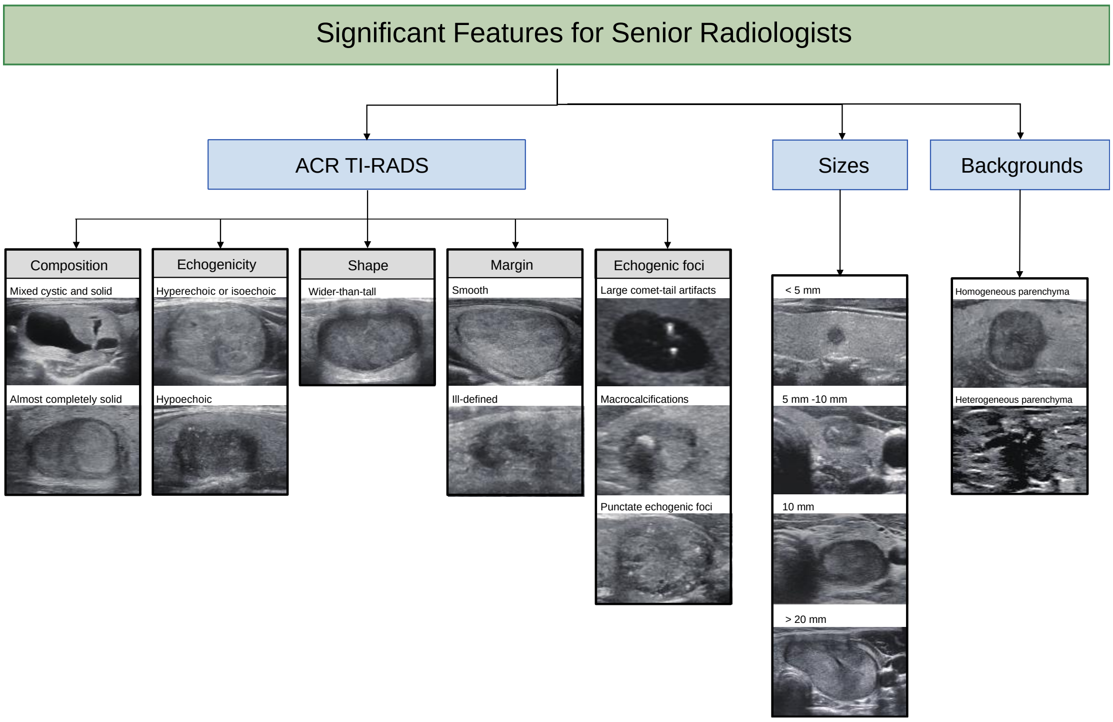

eFigure 2. The Significant Features for Senior Radiologists

For senior radiologists, mixed cystic and solid, solid or almost completely solid, hyperechoic or isoechoic, hypoechoic, wider-than-tall, smooth, ill-defined, none or large comet-tail artifacts, macrocalcifications, punctate echogenic foci, homogeneous and heterogeneous parenchyma, and all nodule sizes were investigated as significant features.

© 2023 Tong WJ et al. *JAMA Network Open.*
