# Supplementary material for: Integration of Artificial Intelligence Decision Aids to Reduce Workload and Enhance Efficiency in Thyroid Nodule Management
Source: JAMA Netw Open. 2023 May 16;6(5):e2313674. doi: 10.1001/jamanetworkopen.2023.13674 (PMC10189570; doi:10.1001/jamanetworkopen.2023.13674)
Supplement: Supplement 2. — Data Sharing Statement [file jamanetwopen-e2313674-s002.pdf]

Data Sharing Statement

Tong. Integration of Artificial Intelligence Decision Aids to Reduce Workload and Enhance Efficiency in Thyroid Nodule Management. *JAMA Netw Open*. Published May 16, 2023. doi:10.1001/jamanetworkopen.2023.13674

Data
Data available: No

Additional Information
Explanation for why data not available: Data supporting the results demonstrated by this study are available within the main text and the Supplementary Information. Other raw data is not shared due to data protection issues. However, we can provide the test for data. For further details the corresponding author may be contacted (wangw73@mail.sysu.edu.cn).
